# A framework to investigate the impact of topography and product characteristics on electronic cigarette emissions

**DOI:** 10.1371/journal.pone.0206341

**Published:** 2018-11-05

**Authors:** Risa J. Robinson, Nathan C. Eddingsaas, A. Gary DiFrancesco, Shehan Jayasekera, Edward C. Hensel

**Affiliations:** 1 Department of Mechanical Engineering, Rochester Institute of Technology, Rochester, NY, United States of America; 2 School of Chemistry and Materials Science, Rochester Institute of Technology, Rochester, NY, United States of America; Battelle Memorial Institute, UNITED STATES

## Abstract

**Significance:**

Protocols for testing and reporting emissions of Harmful and Potentially Harmful Constituents (HPHCs) from electronic cigarettes (e-cigs) are lacking. The premise of this study is that multi-path relationships may be developed to describe interactions between product characteristics, use behavior and emissions to develop appropriate protocols for tobacco product regulatory compliance testing.

**Methods:**

This study proposes a framework consisting of three component terms: HPHC mass concentration, HPHC mass ratio and total particulate mass (TPM) concentration. The framework informs experiments to investigate dependence of aerosol emissions from five electronic cigarettes spanning several design generations and three e-liquids for six repeated trials at each of ten flow conditions.

**Results:**

Results are reported for TPM concentration as a function of flow conditions spanning the range of natural environment topography observed in prior studies. An empirical correlation describing TPM concentration as a function of flow conditions and coil power setting (6, 7.5 and 10 watts) for the Innokin iTaste MVP 2.0 vaporizer with Innokin iClear 30 dual coil tank is presented. Additional results document the impact of flow conditions and wick and coil design on TPM concentration through comparison of the Innokin iClear 30 (upper coil, capillary action wick) and the Innokin iClear X.I (lower coil, gravity fed wick) operated at 7.5 watts. The impact of e-liquid on TPM concentration is illustrated by comparing emissions from an NJOY Vape Pen filled with AVAIL Arctic Blast, Tobacco Row, and Mardi Gras e-liquids. TPM concentration is shown to depend upon flow conditions across a range of e-cigarette product designs including cig-a-like, pen-style, box-mod and emergent disposable-cartridge style devices.

**Conclusions:**

A framework provides a foundation for reporting emissions across a variety of e-cigs, e-liquids and research laboratories. The study demonstrates TPM concentration is a function of topography behavior (i.e. puff flow rate and puff duration) for varying device operating power and product characteristics.

## Introduction

**Fig 1:**
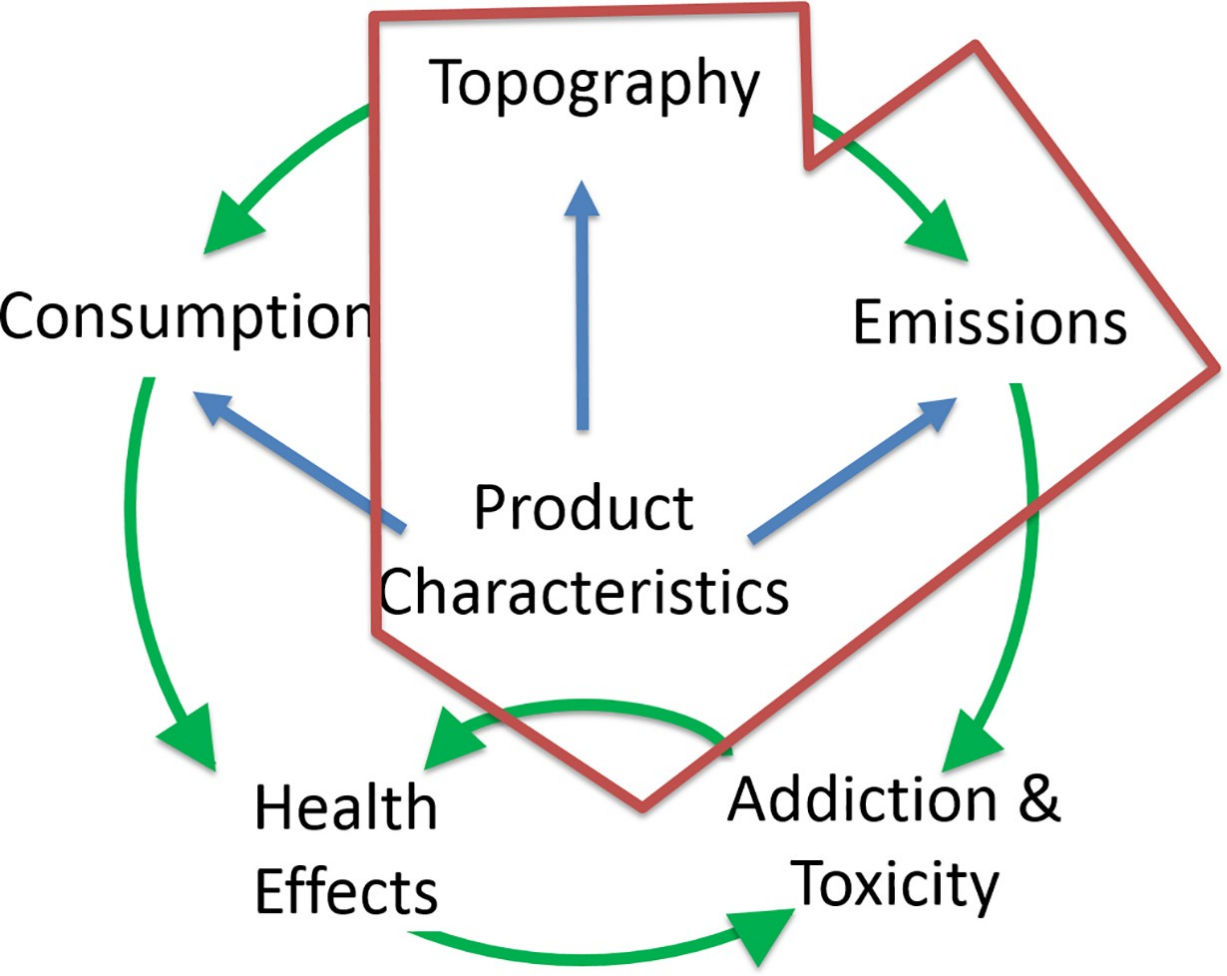
Multi-path relationship between product characteristics, use behavior and emissions. This figure illustrates the premise that characteristics of electronic cigarette designs, e-cig operating conditions (such as power), e-liquid constituents, and user topography behavior (puff flow rate, duration, and volume) jointly impact emissions.

Current United States regulatory guidelines lack specific protocols for testing and reporting the emissions of Harmful and Potentially Harmful Constituents (HPHCs) from electronic cigarettes (e-cigs) (81 FR 28781). Challenges to informing effective tobacco regulatory policy include the variety of e-cig product characteristics (e-cig device designs and e-liquid constituents), rapid market changes in these products, and knowledge gaps in understanding how product characteristics influence user topography behavior, and the manner in which topography behavior and product characteristics affect HPHC emissions, all of which ultimately impact the user’s exposure. The multi-path relationships (**Fig 1**) between product characteristics, use behavior and emissions must be better understood to develop appropriate protocols to guide e-cig and e-liquid manufactures in their testing and reporting of salient product characteristics and the resulting HPHC emissions and exposures.

A wide variety of metrics have been used in reporting e-cig emissions. E-cig “yield” is most frequently reported as the mass of either total particulate matter (TPM) or a constituent (HPHC) captured per session or per puff [1], [2], [3], [4], [5], [6], [7], [8], [9], [10], [11], [12], [13], [14], [15], [16], [17], [18], [19], [20], [21]. As summarized in Table 1, not only do the number of puffs per session vary across studies, but the puff volumes used to generate emission vary across studies. In most studies (but not all), the puff volume and number of puffs are reported such that an “aerosol mass concentration” or the particulate mass per unit volume of air could be inferred allowing for a comparison across studies. However, there would likely be wide uncertainty in such a meta-analysis because studies do not tend to validate the puffing machine settings with an independent flow meter calibrated by a third party. Some studies reported emissions in terms of “mass ratio” or the mass of selected HPHCs per unit mass of TPM [6], [16], [20], [21]. Mass ratio aids comparison across studies but does not lend itself well to incorporating use behavior into risk assessment, because mass ratio does not account for “how much” the user consumes (i.e. puff volume or number of puffs), leading to a potential misinterpretation of the actual amount of exposure. Lack of a consistent framework for reporting emissions outcome measures has led to confusion regarding relative risk of harm from using one product over another.

**Table 1:** Literature e-cig emissions summarizing product characteristics and puff topography studied. Puff flow rate (*q*_*puff*_), puff volume (*V*_*puff*_), puff duration (*d*_*puff*_), e-cig operating power and/or voltage (*W*), e-cig design characteristics (*device*) and e-liquid constituents (*e-liquid*). Shown are the variables tested and the outcome measures used to report emissions. Blank cells indicate that the variable or outcome was not reported.

Study		Puff Volume [mL], (Puff count [–])	Flow Rate [mL/s]	Duration [s]	HPHC Yield [mass HPHC / puff count]	TPM Yield [mass TPM / puff count]	Mass ratio [mass HPHC / mass TPM]
Trehy	2011	100, (30)			device		
Goniewicz	2013a	70, (15)		1.8	device		
Goniewicz	2013b	70, (15)		1.8	device		
Goniewicz	2013c	70, (15)		1.8	device		
Kosmider	2014	70, (15)		1.8	W, e-liquid		
Tayyarah	2014	55, (99)		2	device		device
El-Hellani	2015	NR, (15)	16.7	4	e-liquid		
Farsalinos	2015 IJERPH	55, (100)		4	e-liquid		
Farsalinos	2015 Addiction	NR, (60)			wick design, W		
Jensen	2015	50, (10)		3–4	W		
Talih	2015	68, (15*) 136, (15*) 66, (15*) 132, (15*) 264, (15*)	17 17 33 33 33	4 8 2 4 8	W, e-liquid, V_puff_, d_puff,_ q_puff_	W, e-liquid, V_puff_, d_puff,_ q_puff_	
El-Hellani	2016	NR, (15)	25	4	device, W, e-liquid		
Kosmider	2016	41, 65, 76, (50) 76, (50)		1.8 1.8, 2.7, 3.7		d_puff_, V_puff_	
Kim	2016	NR, (5, 10, 30, 50) NR, (5, 10, 30, 50) NR, (5, 10, 30, 50) NR, (5, 10, 30, 50)	0.83 8.33 16.7 25	2 2 2 2		q_puff_, W, e-liquid***	
Margham	2016	55, (100)		3	V_puff_		
Sleiman	2016	50, (50)	10	5	W	W	device, e-liquid
Kosmider	2017	NR, (74) NR, (47)		5.04^+^ 3.76^+^	d_puff_, e-liquid		
Farsalinos	2017	60, (50)		4	W		
Talih	2017	NR, (3)	16.7	4	device, W	device, W	
Sala	2017	70, (5)		2, 5, 10	device, d_puff_^++^		
Pankow	2017	50, (3 or 6**)		5	device, W, e-liquid	device, W, e-liquid	device, W, e-liquid
Korzun	2018	21, (20) 55, (20)	7.0 18.3	3 3		W, q_puff_	q_puff_

*In the case of high voltage level, 5 puffs were taken and results extrapolated to 15 puffs.

**Not all emissions generated with the same number of puffs. Emissions reported as concentration [μg/m^3^].

***Emissions reported as concentration [mg/L of air].

^+^E-liquid composition and puff duration were not changed independently.

^++^Emissions reported as concentration [μg/mL liquid in the vapor].

Despite the lack of standardized reporting outcome measures for emissions, some important themes have emerged. It is clear that higher power settings increase HPHC yield and mass ratio of many constituents [5], [9], [10], [22] [11], [12], [16], [18], [17], [20] and TPM yield [16], [18], [20]. It has also been reported that e-liquid composition impacts not only the HPHC yield [5]_,_ [7]_,_ [8], [11], [12], [14], [20] and mass ratio [16], [20], but potentially TPM yield [20], [22], [11] as well.

There are limited data available to assess the relationship between topography (puffing behaviors) and emissions. Although several studies report emissions as a function of various topography parameters, as summarized in Table 1, there are limitations in the existing body of literature, supporting the need for more study. Primarily, there is not a wide acceptance for the extent to which puff flow rate influences e-cig emissions, with early studies such as Talih 2015 [11] reporting no impact and later studies such as Kim 2016 [22], Kosmider 2016 [13], Sala 2017 [19], and Korzun 2018 [21] providing evidence to the contrary. Of these studies supporting a potential functionality between puff flow rate and emissions, there are limitations to consider. Although Kosmider 2016 did not explicitly report on puff flow rate, they essentially varied puff flow rate first by holding puff duration constant and varying puff volume, and then by holding puff volume constant and varying puff duration. The authors concluded that emissions depended on puff duration but not on puff volume, thus providing evidence for and against the influence of puff flow rate on emissions. Sala 2017 did not explicitly report a functionality with puff flow rate, but they varied puff duration while keeping puff volume constant, essentially varying puff flow rate, and so the reported conclusion that puff duration impacts emissions, indirectly implies a potential functionality with puff flow rate as well. Korzun 2018 set puff duration constant and varied puff volume in order to achieve two different flow rates. Although they report increased emissions with increased flow rate, these increased emissions appear to be a result of a larger total puff volumes.

In summary, lack of standardization allows for a wide range of test protocols and emissions outcome measures making it difficult to compare products across studies or make inferences about the impact of product characteristics and topography on emissions. There is no clear quantitative understanding of the influence of topography characteristics on emissions, or how topography might influence emissions differently across different products. Flow rate has been cited as an important consideration [21], but only one well-controlled experiment on one e-cig design (vape pen) has been done [22]. Studies are needed to examine the extent to which e-cig emissions are affected by flow rate for the wide range of device types on the market. Power setting, e-liquid composition, and wick design [9] are understood to impact aerosolization effectiveness, but the combined effect of topography, e-liquid, power setting, and wick design has not been investigated and reported in a well-controlled manner.

Herein, we propose a framework that will lay the groundwork for testing and reporting emissions as a function of flow parameters and product characteristics. The framework will enable researchers to generate data based on their laboratory capabilities and research interests, and promote reporting results in a manner that can be better utilized across the tobacco science community. Subsequent meta-analysis of data from different labs would provide a meaningful database to assess differential exposure for emerging products. We apply the framework to analyze the impact of flow rate on emissions across four different device designs and quantify how the functionality is impacted by e-liquid composition, power setting, and wick design.

### Framework

The proposed framework describes e-cig emissions in terms of three emissions outcome measures: total particulate mass concentration (*C*_*TPM*_) of whole aerosol emissions, HPHC mass ratio (*f*_*HPHC*_), and HPHC aerosol mass concentration (*C*_*HPHC*_). This framework is realized mathematically by Eq (1),

$$C_{HPHC}\frac{\left[mg\right]}{\left[mg\right]}=f_{HPHC}(eliquid,topography)\frac{\left[mg\right]}{\left[mg\right]}=C_{TPM}(device,eliquid,topography,power\;setting\frac{\left[mg\right]}{\left[ml\right]}$$ Eq (1)

Where the term “whole aerosol” is taken to encompass all emissions including all species in the gas and particulate phases. The mass concentration, *C*_*TPM*_ [mg/mL], is the total mass of particulates contained per unit volume of air puffed and can be experimentally determined by knowing the mass emissions captured on a filter pad for a measured volume of aerosol passing through the pad. The HPHC mass ratio, *f*_*HPHC*_ *[mg/mg]*, for any single HPHC of interest is computed as the mass of a given HPHC per unit mass of aerosol particulate and can be experimentally determined knowing the mass emissions captured on a pad, and the mass of HPHC molecules quantified by GC-MS analysis of that pad (or other capture media). The subscript ‘HPHC’ represents a single compound, or group of compounds (such as an aldehyde group) of interest to the researcher. While *C*_*TPM*_ is given by a single value for any particular emissions trial under a set of flow and device operating conditions, a separate numerical value of *f*_*HPHC*_ is computed for each constituent. A variety of constituents may be generated which may vary dramatically between flow conditions, device type, operating conditions (such as coil temperature and power), and consumable additives (such as e-liquid flavorants). In some cases, it may be appropriate to quantify these compounds using GC-MS analysis, while in other cases LC-MS or other analytical method may be relevant. The mass ratio *f*_*HPHC*_ is defined relative to the mass of TPM emissions as a convenient and robust base to facilitate reporting of experimental results between research groups. The mass ratio can be determined for decomposition products (such as metals from the coil or furans from flavorings) and for source compounds terms present in the un-puffed e-liquid (such as nicotine and propylene glycol). The HPHC mass concentration, *C*_*HPHC*_ *[mg/mL]*, can be experimentally determined by quantitative GC-MS reported per volume of air passing through the capture media. Again, the subscript HPHC is used to indicate the concentration of any single compound in the emissions. A separate numerical value is computed for each compound under study. For adequate scientific rigor, the aerosol or air volume passing through the media should be measured and reported, in addition to reporting the puff volume based on the emissions generation system settings. *C*_*HPHC*_ is the product of *C*_*TPM*_ and *f*_*HPHC*_; knowing any two of the quantities permits inference of the third. Reporting any one of the quantities yields an incomplete understanding of the relationship between e-cig emissions, product characteristics, and flow condition topography.

Eq (1) is based on the premise that *C*_*HPHC*_ is implicitly a function of product characteristics and topography because each term *f*_*HPHC*_ and *C*_*TPM*_ is presumed to depend on specific product characteristics and flow conditions. Although the functional form of each term is yet unknown, the equation presumes that *C*_*TPM*_ is a function of the e-cig operating power, W, which is supported by the work of others [16], [18], [20], [21]. Eq (1) further hypothesizes that *C*_*TPM*_ is a function of the e-liquid bulk fluid properties (*e*.*g*., saturation temperature, density, and viscosity which are affected by the PG/VG ratio) and topography characteristics (*e*.*g*. puff flow rate, puff duration, and puff volume), although as indicated by the studies listed in Table 1, there is limited data available to support this premise. Baassiri [23] reported that TPM [mg/puff count] and nicotine yield [mg/puff count] are a function of PG/VG ratio. Eq (1) proposes that device design (*e*.*g*. flow path design, wick and coil design and locations) is primarily affecting *C*_*TPM*_ and that *f*_*HPHC*_ is a function primarily of e-liquid components and topography. The current study investigated the functional form of the term *C*_*TPM*_ in the equation. The functional form of *f*_*HPHC*_ is deferred to a future study.

### Objectives

In the current study we aim to quantitatively assess the impact of topography flow conditions (treating puff flow rate, *q*_*puff*_, and puff duration, *d*_*puff*_, as independent variables while holding puff volume, V_*puff*_, constant) on total particulate mass concentration of whole aerosol emissions, *C*_*TPM*_, as a function of product characteristics, including product style (cig-a-like, vape pen, box-mod, and pod styles), power setting, W [W] or [watts], coil/wick design (top or bottom coil design), and e-liquid (three e-liquid flavors).

## Methods

### Apparatus

Devices were mechanically puffed using the PES-1 calibration and emissions system, designed by the Respiratory Technologies Laboratory at the Rochester Institute of Technology (RIT), Rochester, NY, previously introduced in [24]. Suction is provided for the puff via an evacuated chamber maintained at constant vacuum (-25 [kPa] gage for JUUL and -15 [kPa] gage for all other cases). All aspects of puff topography and machine control are under the direction of a National Instruments LabView program and a USB 6008 Multifunction IO. The system operator inputs the flow rate and puff duration as a time series, at a resolution of 0.05 second per time step. The instantaneous flow rate is measured with a calibrated Alicat Scientific flow meter, which provides feedback to a digital control algorithm. The algorithm monitors the puff flow rate and controls a proportioning valve accordingly to achieve the desired puff flow rate, duration, volume and time history. The PES-1 system includes an e-cig power activation unit to depress the power button on tank-type e-cigs. The power activation unit addresses a limitation of previous studies that activated the e-cig manually which limits reproducibility and potential dry puff impact [9, 17]. The activation unit also allows the coil heat-up time to be varied relative to the beginning of a puff.

### E-cig products and components

Five e-cig styles, representing multiple generations were tested (Table 2); blu Disposable (BLU), NJOY Vape Pen (NJOY), Innokin iTaste MVP 2.0 vaporizer with Innokin iClear 30 dual coil tank (i30) and Innokin iClear X.I dual coil tank (iX.i), and a USB rechargeable JUUL (JUUL). All e-cigs were obtained through commercial retailers or on-line e-cig distributors.

**Table 2:** E-cig products, E-liquids, and power settings tested.

Style	Device	Coils/Wick Assembly	Power	Tank Volume	E-liquid (Labeled Nicotine)
Cig-a-like	blu Magnificent Menthol Disposable	2 wicks, absorbent pad	Flow activated	1 [mL]	blu Magnificent Menthol (2.4%)
Vape Pen	NJOY Vape Pen	1 coil, 4 wicks	Button activated, Fixed power	1.6 [mL]	AVAIL Tobacco Row, AVAIL Arctic Blast, and AVAIL Mardi Gras (1.8%)
Box-mod	Innokin iTaste MVP 2.0 with Innokin iClear X.I dual coil tank	2 coils, 1 wick per coil, bottom coil, gravity fed	Button activated, set to 7.5 [watts]	3.0 [mL]	AVAIL Tobacco Row (1.8%)
Box-mod	Innokin iTaste MVP 2.0 with Innokin iClear 30 dual coil tank	2 coils, 4 wicks per coil, top coil, capillary fed	Button activated, varied 6, 7.5 and 10 [watts]	3.0 [mL]	AVAIL Tobacco Row (1.8%)
Pod style	JUUL	1 coil, 2 wicks	Flow activated	0.7 [mL]	JUULpod coolmint (5.0%)

The blu Magnificent Menthol Disposable e-cig contains the e-liquid in an absorbent pad within the atomizer portion of the device. The heating coil is integrated within the atomizer and is supplied with e-liquid via capillary action from wicks that draw it from the absorbent pad as the device is used. Batteries within these devices are non-rechargeable, and have been shown to deplete well before the e-liquid is consumed [24]. Device activation is initiated by an internal pressure sensor that detects the start and end of a puff.

The NJOY is a pen style device having a rechargeable battery and significantly larger capacity relative to the BLU. The 1000 mAh battery was used with the NJOY 1.6 [mL] top fill tank. The atomizer assembly is contained within the removable tank, which is refilled with the user’s choice of e-liquid. The NJOY is activated by pressing a button on the side of the device. There are no user adjustable parameters with this device.

**Fig 2:**
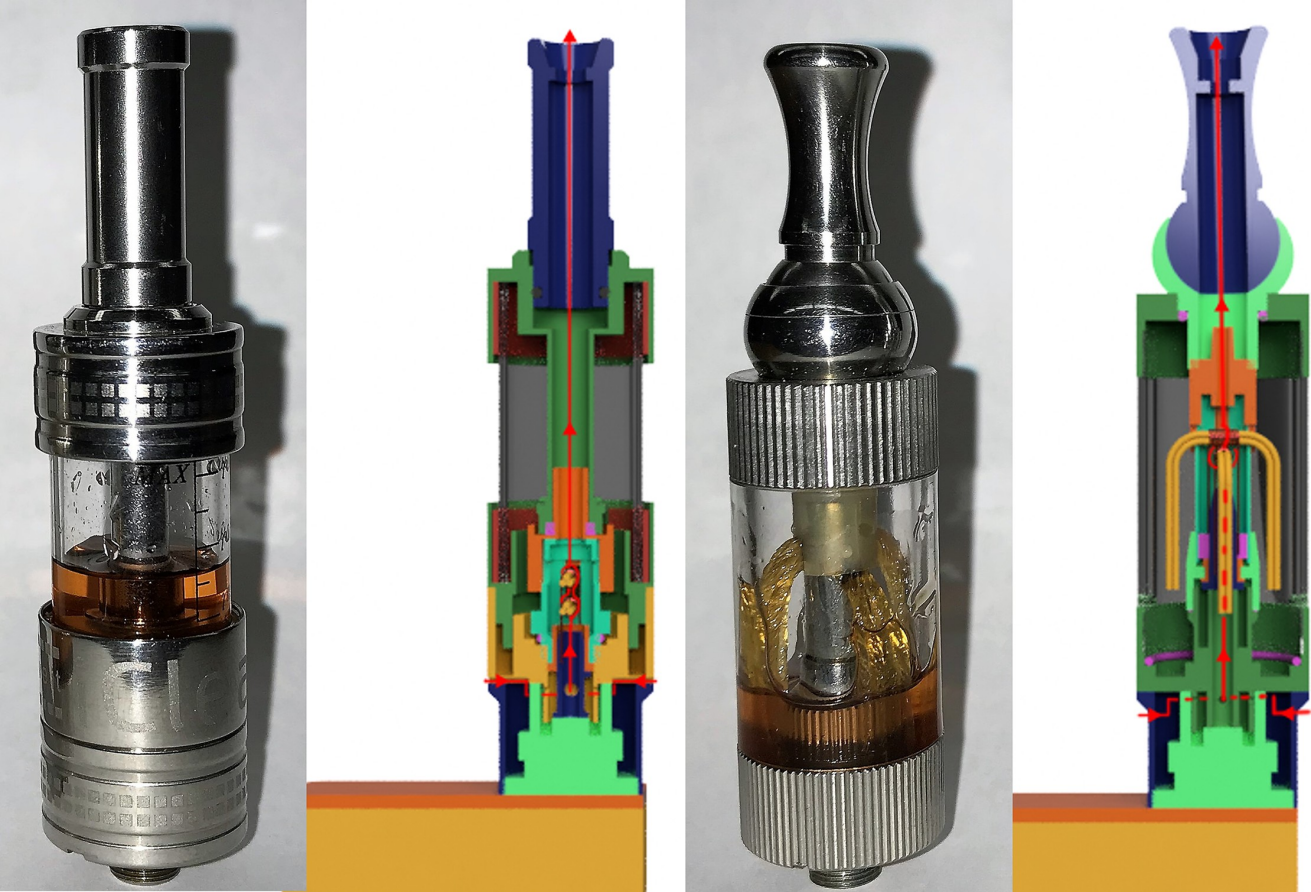
Comparison between the coil/wick designs for the two clearomizer tanks tested in this study. Shown are photographs and CAD renderings created by RIT engineers illustrating the differences in clearomizer designs for Innokin iClear X.I dual coil tank (left two panels) and the Innokin iClear 30 dual coil tank (right two panels). The hypothesis tested in this study is that the bottom coil Innokin iClear X.I with gravity-fed wicks would produce aerosol more effectively (e.g. with higher whole aerosol mass concentrations) than the Innokin iClear 30 upper coil tank with capillary-action-fed wicks.

The Innokin iTaste MVP 2.0 vaporizer is a box-mod having a selectable power range of 6 to 11 [watts] and is a much larger device with a larger battery and larger tank compared to the NJOY. The vaporizer was paired with two interchangeable clearomizer tanks, having significantly different coil and wick designs. As illustrated in Fig 2, the Innokin iClear 30 tank has 2 coils located relatively high in the tank, with 4 wicks per coil extending down into the e-liquid reservoir from both ends (*i*.*e*. 16 total entry points for e-liquid to reach the coil). The Innokin iClear X.I also has 2 coils, but with only 1 wick per coil located at the bottom of the tank. The Innokin iClear X.I wicks are short and constantly immersed in e-liquid during the puffs as long as there is liquid in the tank, whereas the Innokin iClear 30 wicks are significantly longer and rely on capillary action to transport the e-liquid through the wick to the coil.

The JUUL is a hybrid rechargeable power unit with disposable JUULpod cartridges containing e-liquid in free fluid form (not absorbent pads) which are available in a variety of flavors. The JUUL device is flow activated. The heating element and submerged wicks are included in the disposable cartridge, similar to first generation cig-a-like devices, while the power unit is rechargeable, similar to second generation and box-mod devices.

The blu Magnificent Menthol Disposable e-cig was preloaded with a 2.4% nicotine flavored e-liquid. Three e-liquids were tested in the NJOY, including AVAIL Tobacco Row (TR), AVAIL Arctic Blast AB), and AVAIL Mardi Gras MG). The TR, AB, and MG are described on the manufacturer’s web site as “tobacco flavored”, “menthol flavored”, and “fruity mixed berry flavored”, respectively. The label on the packaging of all three e-liquids indicate that they have a 1.8% nicotine concentration. The PG/VG ratio was measured to be 50/50. The Innokin iTaste MVP 2.0 vaporizer was connected to both the Innokin iClear 30 dual coil tank and Innokin iClear X.I dual coil tank which were filled with TR e-liquid. The JUUL was tested in conjunction with JUULpod coolmint e-liquid described as “peppermint flavored” and labeled with 5% Nicotine content.

### Flow parameters

Products were tested for a range of flow conditions (Table 3). Each product was tested with a fixed puff volume of 75 [mL] at 10 different flow rates ranging nominally from 10 to 55 [mL/s] at 5 [mL/s] increments. The resulting nominal puff duration ranged from 1.4 seconds to 7.5 seconds. These topographies are consistent with those previously measured in the natural environment for blu Disposable, [25], a variety of cig-a-likes and NJOY Vape Pen (publication under review). No data are available for box-mod or JUUL device natural environment topography.

**Table 3:** Topography parameters. These are the command topography parameters programmed into the PES-1 system for each e-cig device. Since the device under test impacts the actual flow conditions seen by the device, the actual puff flow rate, duration, puff volume and cumulative case volumes were measured in a closed-loop feedback system. The results and subsequent analyses rely on the actual (measured) rather than the programmed (command) parameters. Each of these 10 cases was repeated for 6 trials for each e-cig product, e-liquid and power setting combinations listed in **Table 2**.

Case	Puff Flow Rate [mL/s]	Puff Duration [s]	Puff Volume [mL]	Number of Puffs	Cumulative Case Volume [mL]
1	10	7.50	75	10	750
2	15	5.00	75	10	750
3	20	3.75	75	10	750
4	25	3.00	75	10	750
5	30	2.50	75	10	750
6	35	2.14	75	10	750
7	40	2.87	75	10	750
8	45	1.67	75	10	750
9	50	1.50	75	10	750
10	55	1.36	75	10	750

### Procedures

Aerosol particulates were captured on a silica fiber filter pad in a Cambridge style filter holder located 4 [cm] from the mouth piece orifice of the e-cig. A new pad was installed before each case of 10 puffs. The filter pad was weighed before and after each set of 10 puffs using a Mettler AE240 analytical balance that had its calibration verified with NIST traceable weights. The e-cig was weighed before and after each set of 10 puffs. In the case of BLU, NJOY, and JUUL e-cigs, the entire device was weighed on the analytical balance. However, in the case of the i30 and the iX.i, the clearomizer tanks were removed from the controller and only the clearomizer tank was weighed. This was necessitated by the capacity limits of the analytical balance. E-cigs were oriented with the mouth piece above the battery, at an approximate angle of 30 degrees from horizontal.

In prior work, BLU e-cigs were found to produce decreasing levels of emission as the battery and e-liquid was depleted from cumulative puffing time [24]. To minimize the impact of decreasing battery capacity, the BLU e-cig trials were performed with fresh units such that no more than 100 seconds of puffing time was experienced by any one unit.

The i30 and iX.i tanks were periodically refilled to ensure that the e-liquid level remained at greater than 50% throughout all trials, to eliminate tank fill as a compounding variable in the analysis. The power activation unit of the PES-1 system was set so that the coil pre-heat time was 0 seconds, to avoid dry puffs [17]. Although no studies are available to inform a realistic pre-activation time, this parameter was controlled and is being reported as part of the experimental protocol.

The JUUL cartridge was monitored to ensure that the e-liquid level remained at greater than 50% throughout all trials, and the cartridges were replaced as needed. The JUUL run time was monitored, and the battery was periodically recharged between cases to ensure that no more than 300 seconds of cumulative puffing time was accrued for each freshly charged battery.

### Analysis

**Fig 3:**
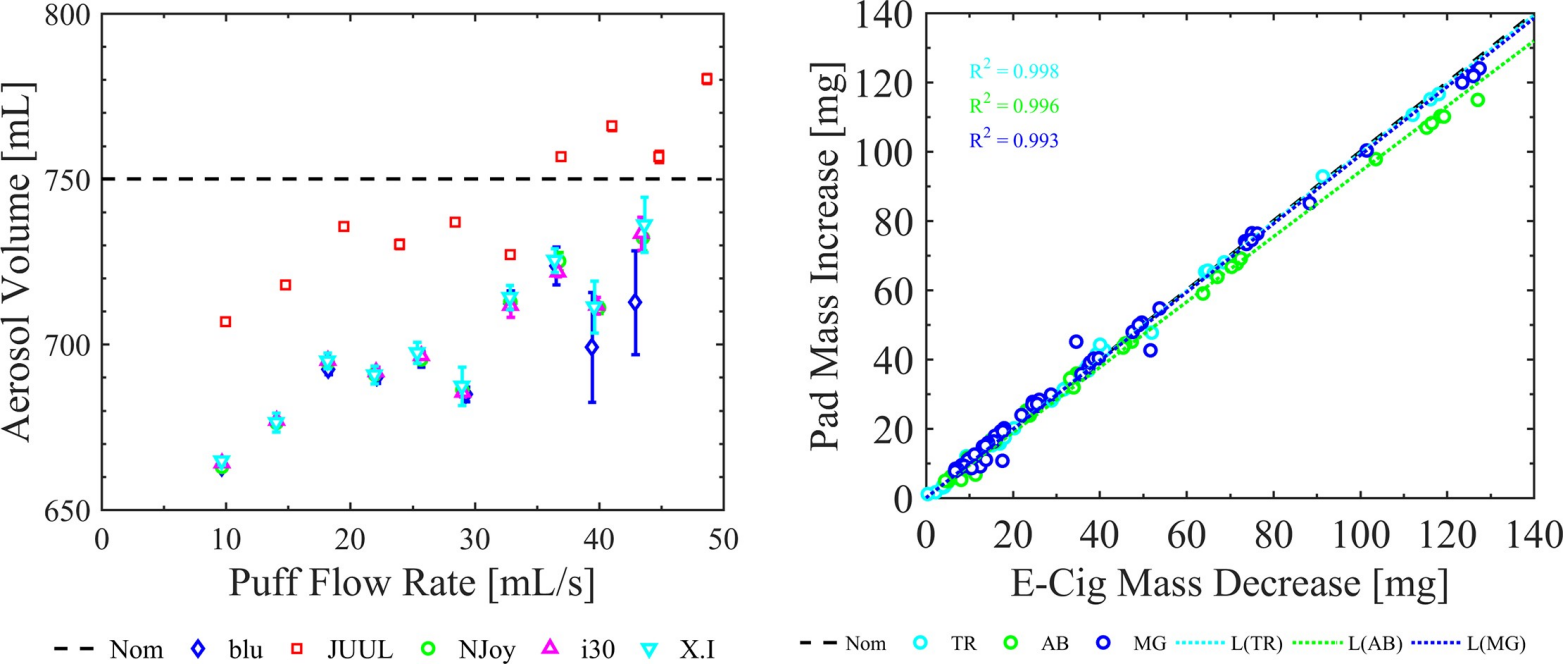
PES-1 System validation plots used to assess the impact of the device under test and the performance of the emission system and verify conservation of mass. Shown are two plots used to validate the PES-1 system for mean measured cumulative volume as a function of mean measured flow rate across six repeated trials at each flow condition for the five e-cigs under test (left), and conservation of mass for various e-liquids (right). The left plot shows that the actual measured volume differed from the nominal machine volume setting for every case. Actual measured volumes and measured flow rates were used in the analysis. The right plot was generated using an NJOY Vape Pen filled with AVAIL Arctic Blast (AB), Tobacco Row (TR), and Mardi Gras (MG) e-liquids across six repeated trials and ten flow conditions. Error bars are the 95% confidence intervals on the mean. Mass conservation was assessed for each product case tested.

The RIT PES-1 emissions system monitored and recorded the actual flow rate achieved throughout a trial. From the measured time dependent flow rate data, the TAP topography analysis program was used to calculate the actual volume of aerosol generated during each trial of each case (Table 3). The measured flow rates and volumes were compared to the command flow rates and volumes as standard operating procedures to assess the maximum operating flow range of each device under test and confirm the prior validation of the emissions system. The observed increase in pad mass was plotted versus the observed decrease in e-cig mass for each trial of each case (Fig 3) to validate conservation of mass in the emissions system. The measured pad mass increase and cumulative puff volumes were used to calculate whole aerosol mass concentration, *C*_*TPM*_ = change in pad mass / measured cumulative volume of air. The mean *C*_*TPM*_ and measured puff flow rate of each case were determined with 95% confidence intervals. The impact of flow conditions on emissions were assessed using plots of total particulate aerosol mass concentration versus measured flow rate. The impact of product characteristics on emissions were assessed by comparing mass emission plots for e-cig generations, clearomizer tank designs, power levels, and e-liquids. Empirical trend lines were assessed as well as emissions at specific flow rates based on the 95% confidence intervals and tests of significance. No emissions data were discarded or omitted from the analysis.

## Results

### System validation

Emission systems must be tuned for each e-cig device under test (e.g. optimize the back pressure as described in the methods section) in order to achieve the desired range of flow rates. Even when emissions systems operate over the desired flow rate range, the actual flow rate will be different from the machine flow rate setting to some degree, the actual puff volume generated will be different than expected, and these differences will vary with flow rate. This is a well understood system dynamics phenomenon that could result in inaccurate concentrations, and inaccurate accounting of flow rate dependence on the outcome measure if not accounted for appropriately. Therefore, we provide results of our system validation and encourage similar transparency and rigor in future emissions publications. The left panel of Fig 3 shows the results of the PES-1 flow loop characterization which was done as part of the system validation. The machine was set to generate ten 75 [mL] puff volumes over the range of flow rates from 10 [mL/s] to 55 [mL/s], yielding a nominal cumulative volume of 750 [mL] per case. In order to achieve this flow rate range, the PES-1 system vacuum tank was operated at -25 [kPa] gage for the JUUL and at -15 [kPa] gage for all other cases. The left panel of Fig 3 provides the actual volumes produced by the emissions system for each e-cig under test, compared to the 750 [mL] nominal machine volume setting across the actual measured range of flow rates. In the case of the JUUL, the volume produced was actually larger than the nominal 750 [mL] set by the machine. This is not unexpected due to the small volume of the JUUL internal flow path and rapid response of the device to the applied suction, which resulted in an overshoot of the commanded flow rate. Because each device under test has a unique dynamic response to the command flow rate, measured volumes and flow rates shown in Table 3 were used in the analysis rather than the machine setting volumes.

The right panel of Fig 3 shows the method used to confirm conservation of mass in the system and assess the potential impact of volatile (gaseous phase) emissions upon whole mass measurements made with filter pads. The horizontal axis represented the measured decrease in the mass of the e-cig between the beginning and end of each trial. The vertical axis represents the increase in the mass of the pad between the beginning and end of the trial. The e-cig characterization shown is for the NJOY, filled with three e-liquids (Table 2). In the ideal nominal case, every data point which lies on the 1:1 line indicates perfect agreement between the mass observations, and thus confirms conservation of mass. Data points above the line suggest mass deposited on the pad is greater than the mass lost from the e-cig, potentially reflecting water vapor condensation from entrained ambient air drawn through the e-cig or simply inaccuracies in mass measurement. Data points below the nominal line suggest that the mass deposited on the pad is less than the mass decreased from the e-cig, suggesting inaccuracies in mass measurement, possible condensation of mass between the exit of the e-cig and the pad surface, or the loss of volatile components passing through the filter pad. Finally, a linear ordinary least squares curve fit was used to assess the slope of the observation data, along with the reported coefficient of determination. A slope significantly less than unity may suggest a significant role of volatile gaseous emissions from the e-cig under certain operating conditions. The validation indicates that the AB e-liquid exhibits a lower slope than either of the TR and MG e-liquids. This observation suggests that analysis of volatiles may yield interesting results for the AB e-liquid.

### Total particulate mass concentration of whole aerosol emissions

**Fig 4:**
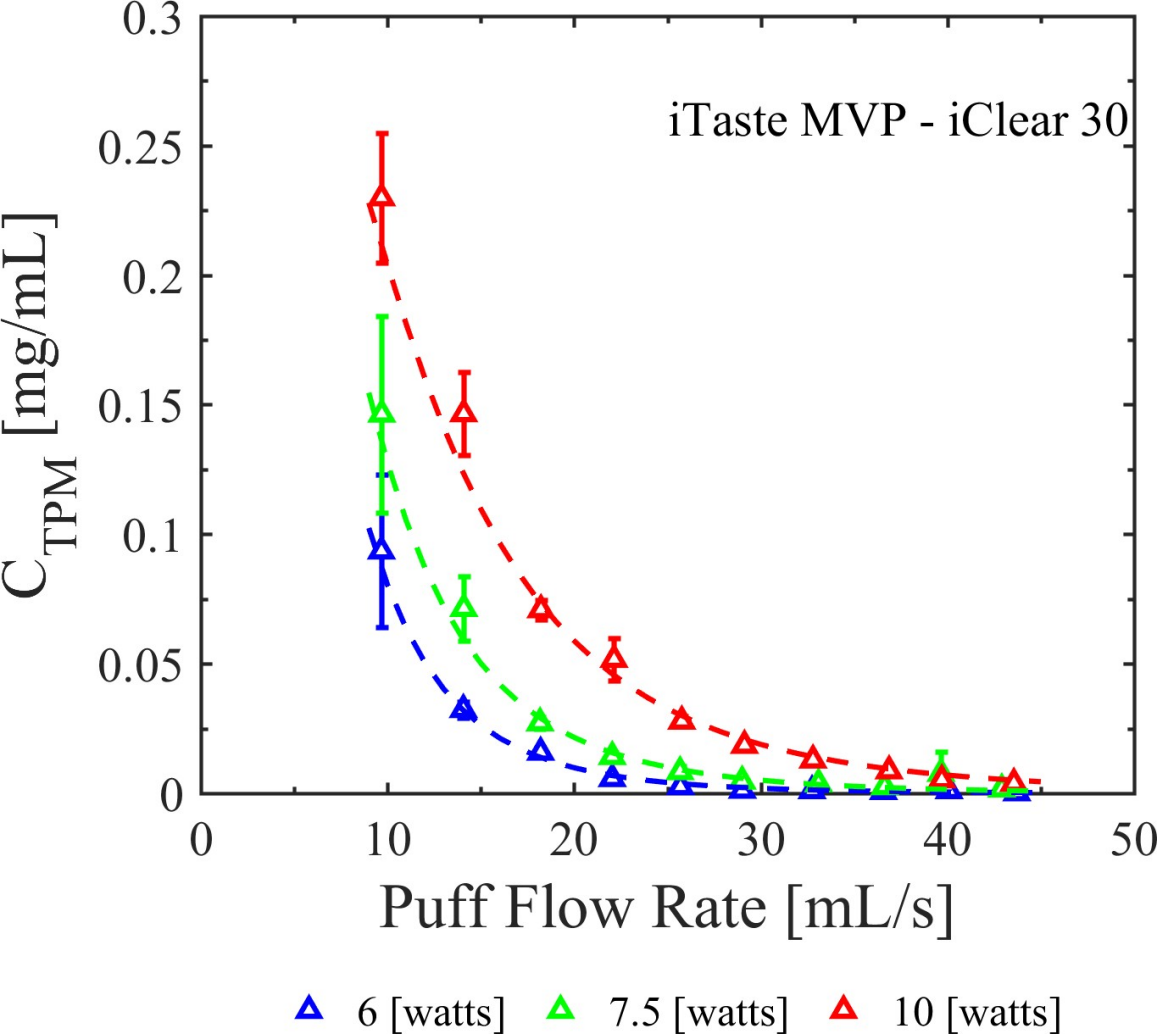
Impact of flow conditions and power setting on total particulate mass concentration of whole aerosol emissions. The Innokin iTaste MVP 2.0 vaporizer with Innokin iClear 30 dual coil tank clearomizer generated aerosols with substantially higher total particulate mass concentrations at lower flow rates, and the flow rate effect was highly dependent on power setting. A correlation equation describing total particulate mass concentration as a function of flow rate and power setting is presented in the text and illustrated as the dashed lines in the figure. Error bars are the 95% confidence intervals on the mean.

Fig 4 illustrates the dependence of *C*_*TPM*_ as a function of flow conditions and coil power setting for the Innokin iTaste MVP 2.0 vaporizer with Innokin iClear 30 dual coil tank clearomizer. Each data point shows the mean *C*_*TPM*_ and 95% CI across six trials at each of ten flow conditions, and three power settings, 6 watts, 7.5 watts and 10 watts. Based on these results, the following empirical model was created to describe the interconnected impact of flow conditions and power level on emissions,

$$\ln\;\left(C_{TPM}\frac{\left[mg\right]}{\left[mL\right]}\right)=-0.8261+0.3085\;(W[w])\;\ln\;\left(q_{puff}\frac{\left[mL\right]}{\left[s\right]}\right)-0.0299\;{\left(W[w]\right)}^{2}-0.9206\;{\ln\;\left(q_{puff}\frac{\left[mL\right]}{\left[s\right]}\right)}^{2}$$ Eq (2)

The model was created using linear regression analysis with a transformation of variables. This model results in coefficient of determination of R^2^ = 0.949 and R^2^_adj_ = 0.943. The functionality of flow rate and power setting on *C*_*TPM*_ is expected to be device specific. Eg. (2) serves as an exemplar model and should not be generalized to other devices without further testing.

**Fig 5:**
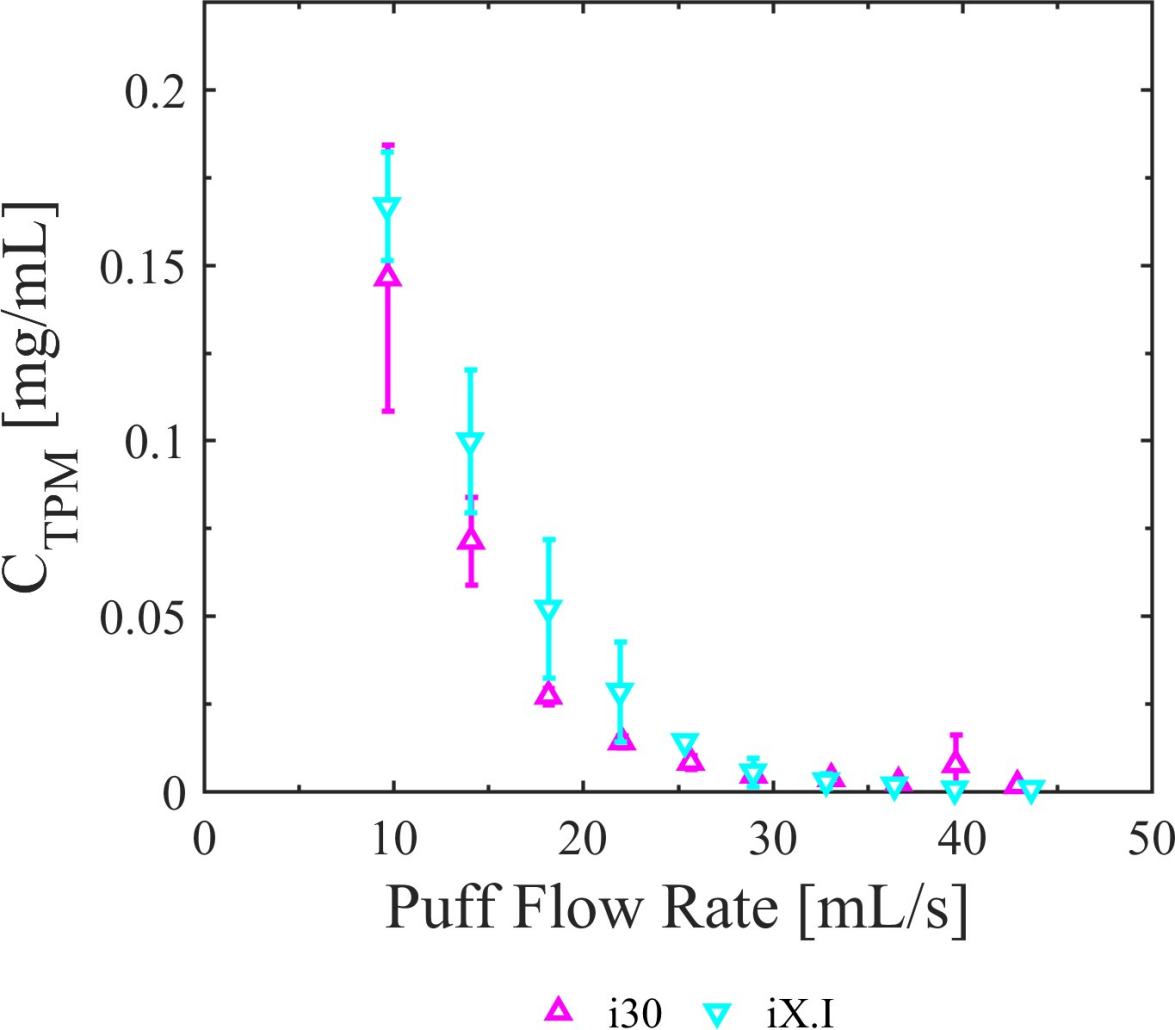
Impact of flow conditions and coil and wick design on total particulate mass concentration of whole aerosol emissions. The Innokin iTaste MVP 2.0 vaporizer with Innokin iClear 30 top coil capillary-fed tank (i30) and Innokin iClear X.I bottom coil gravity-fed tank (iX.I) generated aerosols with higher total particulate mass concentrations at lower puff flow rates and exhibited a dependence upon coil and wick design. Error bars are the 95% confidence intervals on the mean.

Fig 5 illustrates the impact of flow conditions coupled with wick and coil design on *C*_*TPM*_ for the i30 which is a top coil capillary-fed tank and iX.i which is a bottom coil gravity-fed tank, each ran at a power condition of 7.5 [watts]. These results indicate that the bottom coil produces a nominally higher total particulate mass concentration for a given flow condition compared to the top coil design. Increasing the number of repeated trials is anticipated to permit discrimination of significant effects with reasonable power.

**Fig 6:**
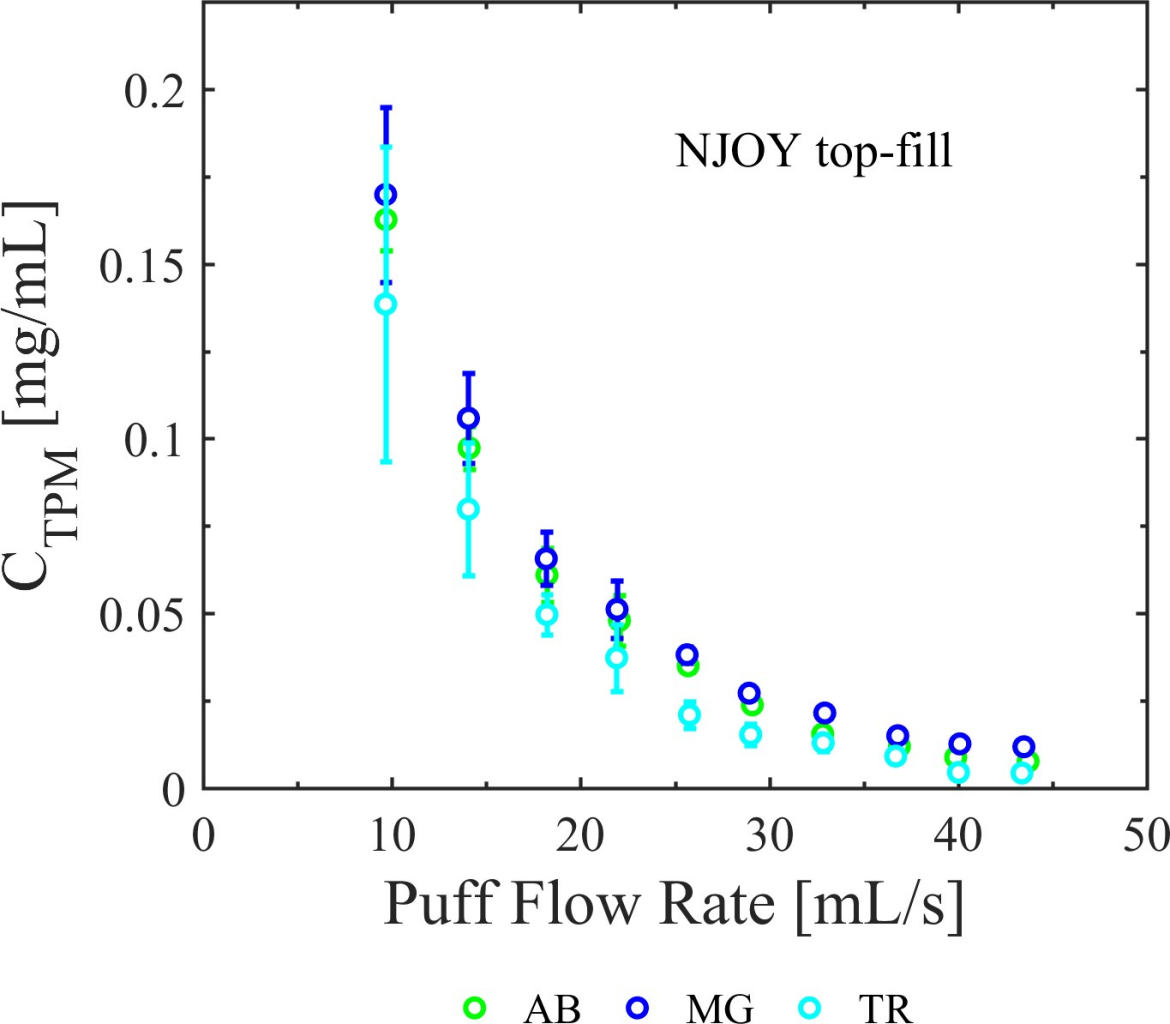
Impact of flow conditions for different e-liquid flavor formulations on total particulate mass concentration of whole aerosol emissions. Shown are emission results for the NJOY Vape Pen filled with three different liquids; AVAIL Arctic Blast (AB), Tobacco Row (TR), and Mardi Gras (MG). Puff flow rate was found to impact the production of aerosol for the three flavors tested and the range of flow rates tested. For all flow rates, the total particulate mass concentration was the highest for MG, followed by AB and then TR. Error bars are the 95% confidence intervals on the mean.

Fig 6 illustrates the impact of flow conditions on total particulate mass concentration of whole aerosol emissions for the NJOY filled with the AB, TR, and MG e-liquids. The results indicate that TR produces aerosol with lower total particulate mass concentration compared to AB and MG for the same flow conditions and that differences are more evident at higher puff flow rates. For example, TR produces aerosol with *C*_*TPM*_ 18% lower than MG at lowest puff flow rate and 64% different at higher puff flow rates. The consistent trends provide a strong foundation to justify further studies, with increased sample sizes, to improve our statistical power and enhance our ability to discriminate the effect of e-liquid composition upon *C*_*TPM*_. The detailed emissions data is available in supplemental data file for Fig 6.

**Fig 7:**
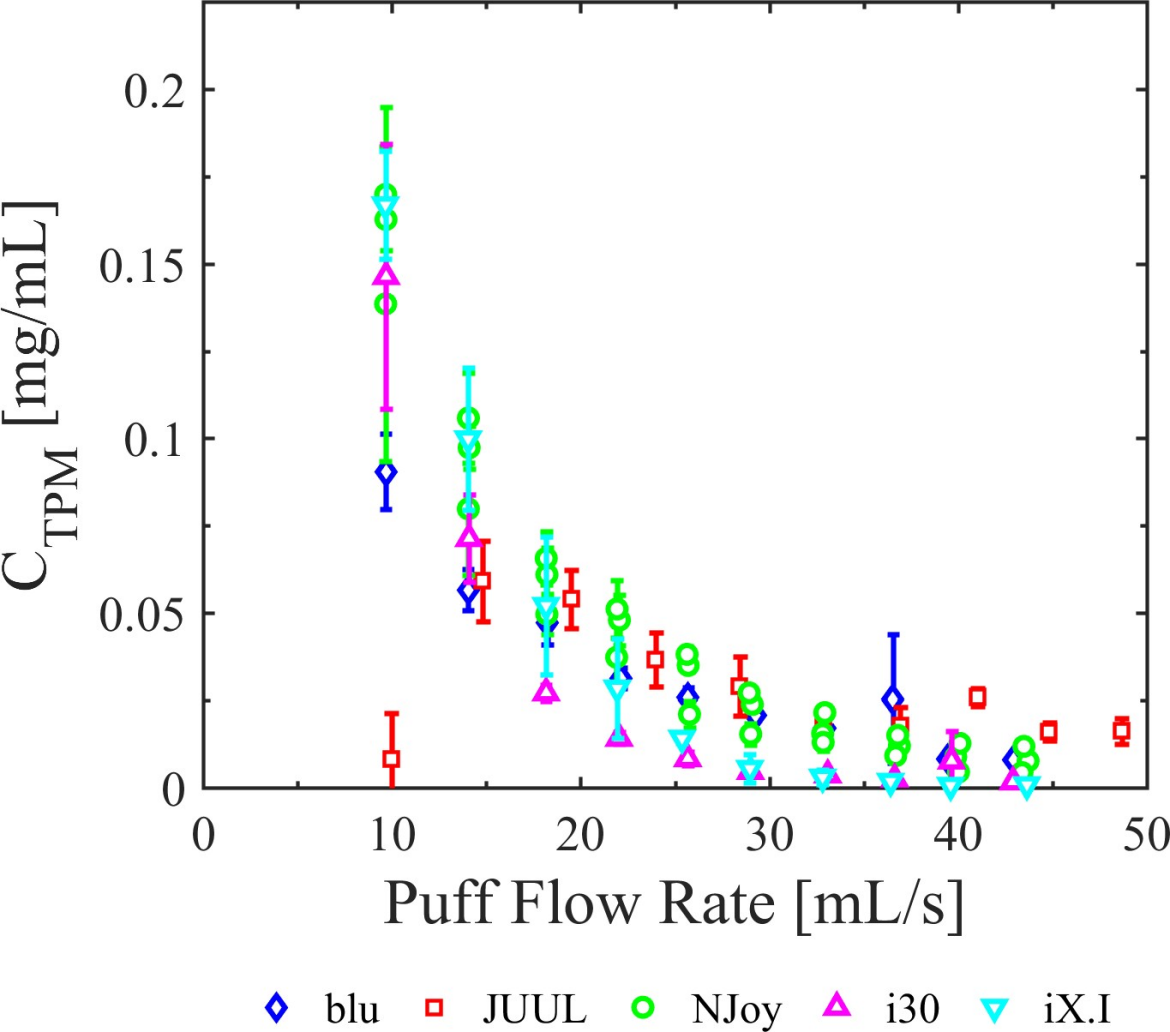
Impact of flow conditions on total particulate mass concentration of whole aerosol emissions across multiple device generations. Flow conditions had a significant impact on total particulate mass concentration for all e-cig generations and styles tested. Shown here are blu Magnificent Menthol Disposable (BLU), NJOY Vape Pen (NJOY), Innokin iTaste MVP 2.0 with Innokin iClear 30 (i30) and Innokin iClear X.I (iX.I), and JUUL (JUUL). Flow rate dependence was more evident for 2^nd^/3^rd^ generation devices (i30, iX.I, NJOY), which have larger tank reservoirs, compared to 1^st^ generation cig-a-likes (BLU) and pod styles (JUUL), which have smaller capacity tanks and exhibited less enhanced aerosol production at lower flow rates. Error bars are the 95% confidence intervals on the mean.

Fig 7 illustrates the impact of flow conditions on total particulate mass concentration over the range of products tested. Flow conditions appear to have a greater impact on larger re-fillable tank style designs (*e*.*g*., i30, iX.i, NJOY) compared to smaller capacity disposable tank style designs (*e*.*g*., JUUL and BLU). The i30, iX.i and NJOY produced total particulate mass concentrations about six times larger at low flow rates compared to high flow rates over the range tested. The flow rate activated JUUL did not reliably produce aerosol at the 10 [mL/s] puff flow rate and was observed to activate only sporadically for this flow condition.

## Discussion

### Flow conditions

Results from this study demonstrate a clear impact of flow conditions across five e-cig products, spanning generations and styles; including cig-a-like disposable (BLU), rechargeable with disposable cartridge (JUUL), pen-style (NJOY), and box-mods with clearomizer (i30 and iX.i). The data consistently support the hypothesis that flow conditions, product characteristics and operating power have a quantifiable impact on the total particulate mass concentration of whole aerosol emissions. The results presented herein were obtained while inversely varying puff duration and puff flow rate to maintain a nominally constant puff volume. Additional emissions results which vary puff flow rate while holding puff duration constant should be presented in a future work.

### Power setting

Consistent with the work of others [16], [20], these data show that coil power impacts whole aerosol mass concentration, *C*_*TPM*_. This is the first study, of which we are aware, to show the additional influence of flow conditions and provide an exemplar empirical predictive relationship accounting for the combined influence of topography and power setting to quantitatively predict *C*_*TPM*_ across a spectrum of power, *W*, and flow rate, *q*_*puff*_, values. The empirical correlation, specific to the Innokin iTaste MVP 2.0 with Innokin iClear 30 tank, strongly supports the dependence of emissions with flow rate and the product of flow rate and power (*p < 0*.*001*). The significance of the power squared term is very strong at low to moderate flow rates up to 38 [mL/s] (*p < 0*.*001*) and degrades with the inclusion of emissions at higher flow rates, (*p ≈ 0*.*01*), attributed to the greater variation in emissions observations at the 7.5 [watt] operating power. All emissions results presented herein were collected using no pre-heating time on the coil. Some users may choose to manually activate devices prior to initiating a puff. Pre-heat time may have a significant impact upon TPM and HPHC emissions, and the impact of pre-heat time on emissions should be addressed in future work.

### Coil and wick designs

The clearomizer tanks tested in this study were chosen to assess the influence of different coil and wick assembly designs on aerosol production. Although both the i30 and the iX.i are dual coil designs, each has unique wetting and airflow implications as a result of their wick design. For example, the shorter gravity-fed wicks on the iX.I are more favorable to wetting compared to the significantly longer capillary-fed wicks of the i30. On the other hand, the i30 has more favorable external flow conditions around the coils. For example, the i30 coil axes are at 90 degrees with respect to one another, whereas the iX.I coils are close together and parallel, with a spacer wick between which impedes flow around the coils and may adversely impact the heat and mass transfer between the aerosol and the heating coil. The coil orientation relative to flow, surface area, e-liquid supply path, operating power, and heat and mass transfer coefficients interact to affect the net TPM emissions from a particular device. The data provide evidentiary support that product design characteristics influence emissions differently at different flow regimes. For flow rates between 10 and 25 [mL/s], at the operating power shown, the *C*_*TPM*_ produced by the iX.I is consistently higher than i30 emissions (p<0.05), indicating the bottom coil wetted wick is favorable to producing highly concentrated aerosols. For flow rates greater than 35 [mL/s], both clearomizer configurations produce comparable emissions, suggesting the benefits of the wetted wick may be balanced out by an adverse flow path associated with coil positioning. This work supports the idea that differences in wick design impact *C*_*TPM*_, and when combined with the framework presented herein, provides additional insight as to why others [9] found wick design to impact *C*_*HPHC*_. These results provide motivation to conduct additional work to better understand the interrelationship between heat and mass transfer mechanisms occurring in the aerosolizer.

### E-liquid

Previous studies have reported variations in HPHC mass ratio, *f*_*HPHC*,_ for different e-liquids [16], [20], but only one previous study reported an impact of e-liquid on total particulate mass concentration, *C*_*TPM*,_ [20]. The current study supports the idea that properties of the e-liquid not only impact *f*_*HPHC*_, but they impact *C*_*TPM*_ as well. The TR e-liquid produced lower C_TPM_ compared to AB or MG (p<0.05). Furthermore, study results show differences in *C*_*TPM*_ as a function of flow conditions, which further supports the idea that bulk fluid properties are a factor in e-liquid whole aerosol production. More work is needed to explore the functionality of e-liquid properties with respect to whole aerosol production.

### Realistic topographies

The flow conditions used in this study represent realistic ranges of topography measured in the natural environment for blu [25], cig-a-likes [26] and NJOY (study not yet published). The study results are limited by not knowing the topography for box-mod style and pod style devices. However, the topography parameters tested do span the range of user behavior anticipated for these devices. Once topography data are available, the current study can be extended to apply the additional flow parameters if they are indeed outside the range tested here.

### PES-1 emissions system validation

It is unclear whether or not the dynamic response of emissions systems used to generate e-cig aerosol has been considered in the published work of others. It is common to report total mass collected for a reported number of fixed-volume (machine-setting) puffs, rather than to report a concentration based on measured volumes. Such reporting trends result in wide uncertainties with respect to the conditions under which the emissions were generated, and impede our ability to uncover the actual functionality of the emissions with respect to topography parameters. The validation done in the current study clearly demonstrates that the puff flow rates and volumes produced by the emissions system may not equal to the nominal machine settings. The magnitude and statistical significance of this interaction are jointly a function of the emissions system and the e-cig under test. This interaction is supported by Fig 3 for the devices tested in this study. The 95% confidence intervals demonstrate the actual volume is statistically different than the nominal for the flow conditions and products tested. Actual (measured) puff flow rates and puff volumes were used in all results herein to eliminate potential bias due to dynamic response of the emission system. The current work serves to demonstrate the importance of reporting emissions test results in terms of measured flow rates and measured aerosol volumes, and that it is insufficient to rely upon nominal “machine settings” of emissions systems.

### Yield

Several previous articles have reported emissions results in terms of yield, often with no clear indication of the puff duration, puff flow rate, puff volume and puff count used to measure the yield, often reported as [mg/puff]. This lack of consistency in reporting may lead to inaccurate conclusions regarding the dependence of emissions upon flow conditions. The framework presented herein is directly focused on this challenge and conclusively demonstrates the necessity of reporting all flow and operating conditions associated with emissions reports. Results presented herein conclusively demonstrate that TPM yield expressed as [mg/puff] does indeed vary as a function of flow conditions. The design of experiments (Table 3) assured that each emissions trial had nominally the same per puff and cumulative session volume. Thus, one may multiply the vertical axis of any C_TPM_ figure (Figs 4 through 7)by a constant scale factor to interpret these figures as TPM yield on either a per-puff [mg/puff] or per-session [mg/session] basis. Clearly, TPM yield and concentration are both dependent upon flow conditions. The inconsistency in prior reporting, particularly yield per puff [mg/puff], may lead to inaccurate interpretation of the mechanisms of e-cig operation.

## Conclusions

This paper proposes a framework expressing the HPHC mass concentration, *C*_*HPHC*_, as the product of the HPHC mass ratio, *f*_*HPHC*_, and the whole aerosol total particulate mass concentration, *C*_*TPM*_. The framework provides a consistent foundation for reporting emissions testing results across a variety of electronic cigarettes, e-liquids and research laboratories. The framework permits investigators to study *f*_*HPHC*_ of HPHCs of interest to evaluate the impact of e-liquid composition for example, while reducing the need to assess *C*_*TPM*_ across device generation. Similarly, other research teams may investigate the impact of changes in device product characteristics to assess their impact on *C*_*TPM*_.

Whole aerosol mass emissions, *C*_*TPM*_, have been conclusively demonstrated to be a function of user behavior (reflected by puff flow conditions), user-choice regarding device operating power (reflected by device power settings, or the surrogates of voltage and temperature) and product characteristics (flow path, coil and wick location, e-liquid composition, *etc*.). We demonstrated the ability to create an empirical correlation of the dependence of whole aerosol mass emissions as a function of these parameters for a device. Further work is required to extend the predictive capabilities of the model to a broad range of products and operating conditions, and to investigate the functionality of *f*_*HPHC*_. Emissions tests which independently vary flow rate and duration are needed to provide a clearer picture as to which variables are most influential. We recommend that experimental emission studies adopt the protocol of using and reporting measured flow conditions rather than machine settings, because such scientific rigor will reduce uncertainties, support comparison across studies and improve the likelihood that statistical correlations can be discovered.

Understanding the multiple interconnected pathways between product characteristics, user topography and HPHC mass concentration will lead to a more sophisticated understanding of tobacco product emissions data, having implications on reducing the number of variables required to test e-cig emissions to inform tobacco regulatory policy.

## Supporting information

S1 Dataset Underlying data for Fig 3, validate volume. (CSV)

S2 Dataset Underlying data for Fig 3, validate mass. (CSV)

S3 Dataset Underlying data for Fig 4, emissions by power setting. (CSV)

S4 Dataset Underlying data for Fig 5, emissions by coil design. (CSV)

S5 Dataset Underlying data for Fig 6, emissions by E-liquid. (CSV)

S6 Dataset Underlying data for Fig 7, emissions by device generation. (CSV)
